# Calcium and Vitamin D Supplementation as Non-Surgical Treatment for Periodontal Disease with a Focus on Female Patients: Literature Review

**DOI:** 10.3390/dj10070120

**Published:** 2022-07-01

**Authors:** Zana Sllamniku Dalipi, Fatmir Dragidella

**Affiliations:** Periodontology and Oral Medicine Department, University Dentistry Clinical Center of Kosovo, Medical Faculty, University of Prishtina, Street Lagjja e Spitalit p.n., 10000 Prishtina, Kosovo; zana.sllamniku@uni-pr.edu

**Keywords:** vitamin D, calcium, periodontal disease, dysbiosis, women pregnancy, maternal health

## Abstract

Periodontal disease is a complex disease that involves an imbalance between the oral microbiota and an individual’s inflammatory response. Moreover, the inflammatory response contributes to further imbalance; if left untreated, periodontal disease may result in tooth loss. Vitamin D is intricately involved in the regulation of calcium–phosphate homeostasis and bone mineral metabolism; considering that periodontal surgery usually includes regenerative therapy, adequate vitamin D and calcium levels are essential. The benefits of vitamin D and calcium supplementation have been demonstrated in situations where deficiency is associated with adverse outcomes, such as periodontal disease and maternal health. However, knowledge and attitudes about supplementation, as well as the actual levels of supplementation, vary greatly in the general population; they also differ between men and women. The aim of this review is to discuss how vitamin D and calcium supplementation affect oral and periodontal health, especially in women. Additionally, this review provides suggestions for public health strategies regarding vitamin D and calcium supplementation, as well as the effects of both types of supplementation on maternal oral health.

## 1. Introduction

Periodontal disease is a complex polymicrobial disease, which involves the disruption of oral homeostasis and is characterized by gum inflammation (gingivitis) that leads to progressive loss of tooth-supporting tissues [1,2]. This disease results from an imbalance between the oral microbiota and an individual’s inflammatory response; it contributes to dysbiosis [3]. The systemic effects of the host response to periodontal disease are suspected to develop along with dysbiosis, resulting in a state of nososymbiocity [4].

Periodontal disease has important systemic effects and can exacerbate other diseases [3,5,6]. These effects are partly mediated by the movement of pro-inflammatory cytokines from oral tissues into systemic circulation. In the liver, these cytokines increase the levels of various proteins (e.g., C-reactive protein, fibrinogen, and serum amyloid A) that reportedly exacerbate atherosclerosis and intrauterine inflammation. Additionally, periodontal bacteria can directly enter systemic circulation during tooth brushing or tooth extraction [7], producing at least 5 min of detectable bacteremia [8].

The major predictors of tooth loss in patients with periodontal disease include the presence of plaque-associated bacteria, older age, poor compliance with dental care, smoking, and diabetes [1,4]. Vitamin D and calcium supplementation can have a positive effect in the management of periodontal disease, when used as an adjunct to non-surgical periodontal treatment; moreover, vitamin D and calcium supplementation may reduce tooth loss and alveolar ridge resorption [9,10].

Although guidelines have been published, there is a lack of consensus among countries regarding non-surgical periodontal therapy. For example, articles discussing guidelines were published by Imrey et al. in 1994 [11] in the USA and by Vandenbulcke et al. in 2008 [12] in Belgium. Imrey et al. [11] indicated that the proposed guidelines were meant to provide a framework for future development (i.e., the authors proposed a method for evaluating adjunct therapies, rather than stand-alone guidelines for direct clinical application), whereas the article by Vandenbulcke et al. [12] specifically described guidelines for non-surgical periodontal therapy in Belgium. Furthermore, the American Academy of Periodontology published limited guidelines (without extensive explanation) in 2001 [13] and the American Dental Association published comprehensive guidelines (with information regarding level of certainty, adverse effects, and strength of recommendation) in 2015 [14]. A recent report by Könönen et al. [15] compared recommendations by the European Federation of Periodontology with guidelines in Nordic countries; the Nordic guidelines were generally in agreement with recommendations by the European Federation of Periodontology. Notably, the European Federation of Periodontology recommendations discussed clinical treatment and risk factor control, while the American Dental Association guidelines focused entirely on clinical treatment; there were also differences in guidance concerning sub-antimicrobial dose doxycycline and non-surgical use of lasers as adjunct therapy. Additionally, the *Journal of Clinical Periodontology* published a series of articles regarding clinical guidelines for the treatment of periodontal disease in 2020 [16]; some of the systematic review findings differed from the clinical practice guidelines on the same issue (e.g., concerning the use of systemic antimicrobials during periodontal therapy).

The host inflammatory response is involved in periodontal disease; therefore, adjunctive methods to mechanical non-surgical periodontal treatment are needed in some patients. In addition to vitamin D and calcium, multiple newer non-surgical modalities for periodontal disease have been investigated. These non-surgical modalities include probiotics, prebiotics/synbiotics, statins, pro-resolving mediators, omega-6 and -3 therapy, ozone therapy, and epigenetic therapy [17], as well as photodynamic therapy (specifically as an adjunct therapy) [18].

Although studies have evaluated the potential for vitamin D and calcium to prevent alveolar bone loss and improve periodontal disease, only two randomized controlled trials (RCTs) have been published to date [19,20]. Furthermore, sex differences have been reported in vitamin D and calcium status [21] and supplementation; previous studies have not addressed these differences [22,23,24]. In the literature, the effects of vitamin D and calcium supplementation in men and women have been described together without consideration of any sex differences, despite evidence that the potential to develop periodontal disease is affected by pregnancy-related hormonal changes that impact oral health [25]. Moreover, periodontal disease has been associated with preterm birth [26] and/or low birth weight [27,28], which is associated with an increase in neonatal mortality rate [29].

The aim of this review is to discuss the aspects of vitamin D and calcium supplementation that affect oral and periodontal health, with a focus on women, including pregnant women. Additionally, this review provides suggestions for public health strategies regarding vitamin D and calcium supplementation, as well as the effects of both types of supplementation on maternal oral health.

## 2. Methods

A narrative review search strategy was used to locate the relevant literature on the effect of vitamin D and/or calcium supplementation on oral and periodontal health, especially related to female patients. For this literature review, we searched PubMed from 1994 onwards using the MeSH terms “vitamin D,” “calcium,” “oral disease,” “periodontal diseases,” “pregnancy,” and “maternal health.” Bibliographies of identified manuscripts were also reviewed for additional articles of interest.

Both authors (ZSD and FD) considered all types of peer-reviewed and full-length studies in English. In total, the literature review found 336 papers, of which 157 were identified as being of interest after reading the title and abstract and removing duplicates. This included RCTs (n = 7), observational studies (n = 71), retrospective studies (n = 56), meta-analyses (n = 4), systematic review articles (n = 12) and other types of reviews (n = 27).

For the guiding principles of this review, we used the critical appraisal tool SANRA (Scale for the Assessment of Narrative Review Articles) to control for the quality of this manuscript [30]. The SANRA tool was developed to formally critique the originality and importance of the topic to the reader, as well as the scientific reasoning behind key arguments, and the review’s appropriateness and completeness. In brief, the SANRA tool covers the following topics: (1) explanation of the review’s importance/relevance, (2) statement of the aims of the review, (3) description of the literature search, (4) targeted referencing, (5) solid logic or scientific reasoning and (6) adequate presentation of relevant and appropriate endpoint data, also with conclusions.

## 3. Theory Supporting Vitamin D and Calcium Supplementation as Non-Surgical Treatment for Periodontal Disease

### 3.1. Key Physiological Roles of Vitamin D and Calcium

**Figure 1:**
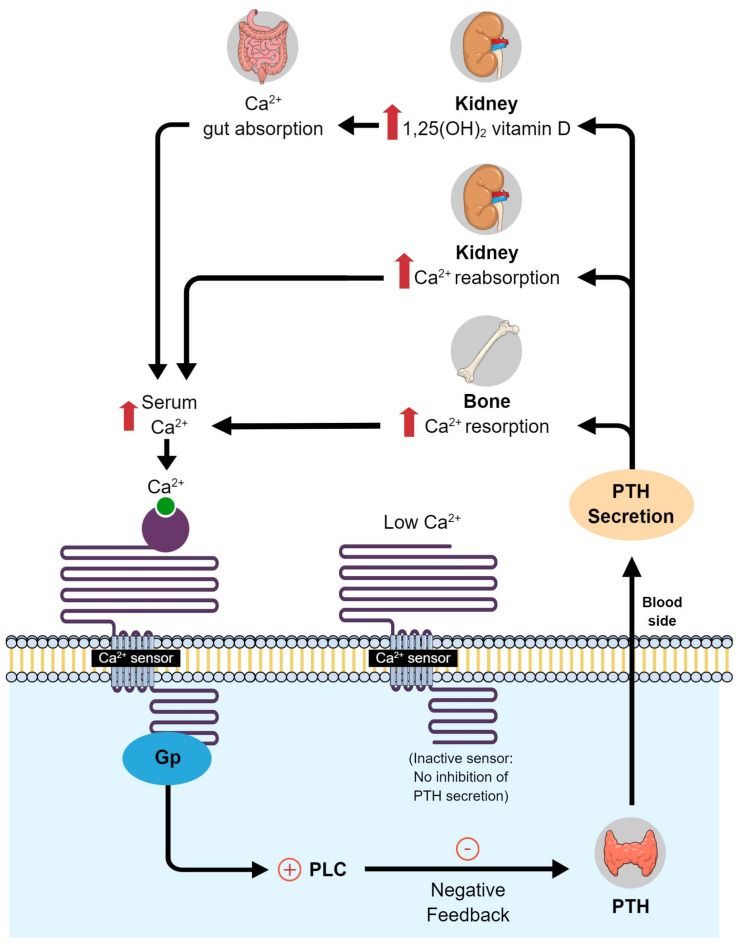
Calcium and vitamin D metabolism in the body. PLC, phospholipase C; PTH, parathyroid hormone.

Vitamin D is a key factor in the regulation of calcium–phosphate homeostasis and mineral bone metabolism [31]. This vitamin increases absorption of calcium in the intestine and decreases parathyroid hormone secretion, thereby reducing systemic bone resorption (Figure 1). Vitamin D also stimulates osteoclasts and alkaline phosphatase activity, optimizes bone remodeling, and affects bone mass by increasing bone matrix protein production [31]. Thus, vitamin D has important roles in bone metabolism. Because periodontal surgery usually involves bone regeneration [32], adequate vitamin D levels are essential.

### 3.2. Relationships of Serum Vitamin D and Calcium Levels with Systemic and Periodontal Inflammation

Various benefits of vitamin D and calcium supplementation have been reported [33,34,35]. For example, Meghil et al. [34] supplemented 23 patients with 25-hydroxyvitamin D (OH)D (25[OH]D), 4000 IU orally, for 12 weeks. The authors measured serum 25(OH)D levels before and after supplementation; they found a two-fold increase after supplementation, compared with baseline levels. In the same study, the authors observed decreased blood levels of inflammatory markers and salivary pro-inflammatory cytokines. Furthermore, analysis of peripheral blood mononuclear cells revealed that the levels of antimicrobial autophagy-related proteins were higher in participants who had received supplementation. However, the authors reported that supplementation did not have a significant effect on clinical parameters, presumably because the patients did not exhibit vitamin D deficiency at the onset of supplementation. Bonnet et al. [36] analyzed Canadian Health Measures Survey data for 5604 participants aged 13–79 years to evaluate the association between 25(OH)D levels and periodontal disease. Periodontal disease was evaluated by the gingival index and loss of attachment. The authors concluded that there was modest evidence to support a relationship between 25(OH)D levels and periodontal disease; specifically, patients with lower 25(OH)D levels exhibited greater loss of attachment and a more severe gingival index. In addition to its effects on bone metabolism, a study of gingivitis severity indicated that vitamin D has a dose-dependent anti-inflammatory effect [20].

### 3.3. Vitamin D and Calcium Supplementation in the General Population

Today, factors that complicate achieving adequate internal levels of vitamin D and calcium are the lower levels of sun exposure and calcium intake, compared with previous generations [37]. A specific threshold of serum 25(OH)D for vitamin D deficiency has not been established [38,39]; moreover, it has been difficult to establish recommendations for optimal levels [40]. The Endocrine Society Task Force on Vitamin D has defined <75 nmol/L as the threshold for vitamin D deficiency, while other groups use thresholds of <25 or <30 nmol/L because these levels are associated with increased risks of osteomalacia and rickets [40,41]. However, even for the lowest threshold of 25(OH)D (i.e., <25 nmol/L), there is consensus in both low- and high-income countries regarding the need to address vitamin D deficiency [38]. It is challenging to address this issue for a few reasons, which are as follows: environmental and individual factors affect 25(OH)D generation from sun exposure; naturally rich sources of vitamin D are scarce and may be consumed infrequently; and nutritional surveillance data indicate that vitamin D intake is generally lower than the recommended level [38].

The sources of vitamin D are sun exposure, fatty fish, and oral supplementation [42]. There is reportedly limited knowledge in the general population regarding the health benefits of vitamin D. For example, several studies revealed that participants were unsure about which foods are good sources of vitamin D [24,42,43]. Participants in another study [43] were confused by unclear messaging from health professionals regarding the risks and benefits of sun exposure. Additionally, a study of Canadian university students found that only 14% knew the correct amount of time required in the sun (10–30 min of mid-day sun several times per week) to generate vitamin D in the skin [44].

Although sun exposure is the main source of vitamin D, there are multiple barriers to sun exposure; these include climate, living in high-rise buildings, limited access to outdoor public spaces, low physical activity, and cultural customs (e.g., wearing clothing that covers most or all of the skin) [24]. Thus, there is a critical need for knowledge regarding the importance of supplementation. However, there have been mixed results regarding attitudes towards vitamin D supplementation [24,42,45,46,47]. Supplementation was considered harmful/helpful, and/or ineffective, depending on the study. Several factors are associated with the barriers to vitamin D and calcium supplementation, including the unpalatability of combined tablets [44].

## 4. Effects of Vitamin D and Calcium Supplementation on Periodontal Disease

**Figure 2:**
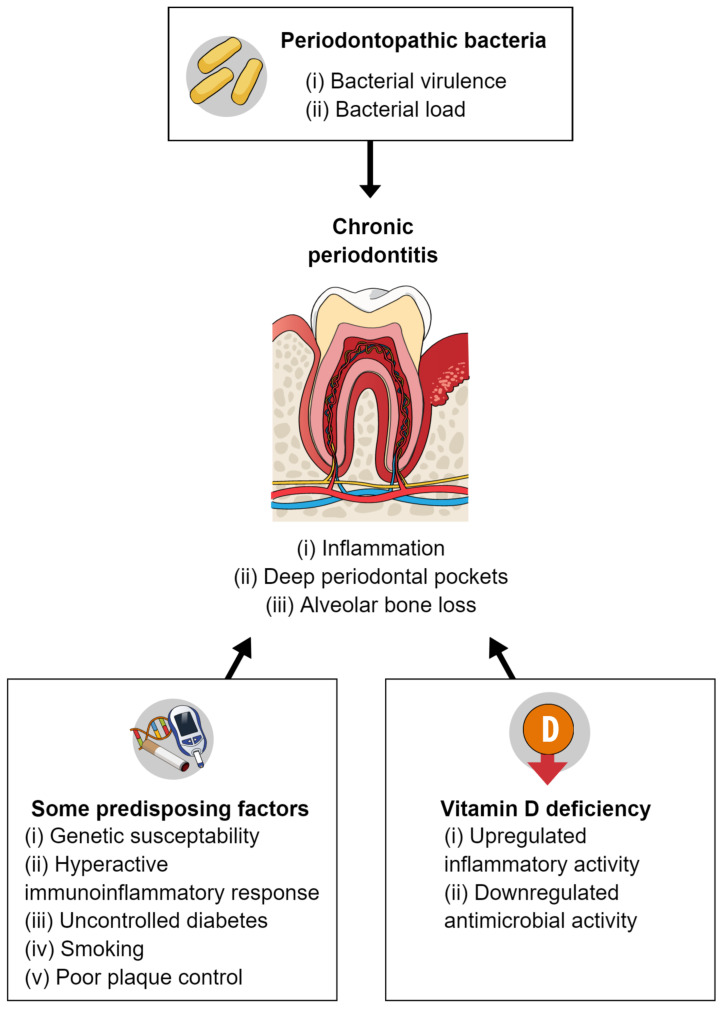
Pathogenesis of periodontal disease and a possible relationship with vitamin D deficiency.

The effects of vitamin D and calcium supplementation on periodontal disease have not been fully determined (Figure 2). Most published studies concerning vitamin D and periodontal disease are observational, with cross-sectional or case–control designs; thus, they have limitations compared with studies such as RCTs. Studies concerning calcium and periodontal disease are also generally observational; nearly all have had a cross-sectional design [48]. Furthermore, the required dose of vitamin D varies according to population (e.g., healthy adults vs patients with illness) and season (e.g., higher doses/greater supplementation is needed in winter). The required dose also depends on an individual’s pre-supplementation levels of 25(OH)D. Vitamin D supplementation at appropriate doses is generally considered safe, although doses above the recommended amounts can be harmful. The effects of calcium supplementation on periodontal disease may be influenced by the presence of sufficient vitamin D and protein [48].

Pinto et al. [49] conducted a systematic review to explore whether patients with lower vitamin D levels have an increased risk of periodontal disease; they also explored whether treatment outcomes are improved during supplementation or in patients with elevated levels of serum vitamin D. The authors identified 27 studies, including 13 cross-sectional, 6 case–control, 5 cohort, 2 RCTs, and 1 case series. Among the cross-sectional studies analyzed, 65% indicated that low vitamin D levels were significantly associated with periodontal disease. However, the findings highlighted the need for additional rigorous studies with standardized definitions for periodontal disease and internal vitamin D levels. Additionally, the findings of the only two published RCTs [19,20] to date suggest that vitamin D supplementation can aid in the prevention of tooth loss and gingival bleeding. One of the RCTs [19] evaluated vitamin D and calcium supplementation in adults aged ≥65 years over a 3-year period; 13% of the supplemented patients lost teeth, compared with 23% of the non-supplemented patients. In the other RCT, Hiremath et al. [20] reported a dose-dependent anti-inflammatory effect of vitamin D after supplementation at a dose of 2000 IU/day, 1000 IU/day, or 500 IU/day. All three groups showed significant (*p* < 0.0001) improvement in gingivitis based on gingival scores; improvement occurred more quickly at the higher doses. However, the extent of this improvement in gingivitis is unclear. A recent study by Gao et al. [35] evaluated attachment loss and probing depth in patients who received 2000 IU/day vs 1000 IU/day vitamin D or vs placebo. Although the authors found a statistically significant difference between vitamin D supplementation and placebo in favor of supplementation, they concluded that the magnitude of the effect was modest and had limited clinical relevance.

Van der Putten et al. [50] performed a systematic review of studies that evaluated the associations of deficiencies in vitamin B complex, vitamin C, vitamin D, calcium, and magnesium with periodontal disease in older adults. The authors found no conclusive evidence to support associations of such deficiencies with periodontal disease in older adults. Amarasena et al. [51] evaluated the progression of periodontal disease by measuring attachment loss in 266 Japanese non-institutionalized participants aged 70 years. The authors measured serum calcium, albumin, random blood sugar, immunoglobulins (IgG, IgA, and IgM), sex, smoking habits, education, gingival bleeding, and the number of teeth present at baseline. Serum calcium was the only variable significantly associated with the progression of periodontal disease. In a systematic review, Perić et al. [52] found that some data from the analyzed studies supported a “perio-protective” role for vitamin D; however, they did not evaluate calcium in that review.

Several systematic reviews have assessed serum 25(OH)D levels in patients with chronic periodontal disease [49,50,51,52,53,54]. The populations examined in these studies varied from a narrow focus (e.g., patients with chronic periodontal disease vs healthy controls [54]) to a broad focus (e.g., any study (human or animal) evaluating the relationships of vitamins with periodontal disease [53]). For example, Machado et al. [54] analyzed the findings of 16 studies involving 10,597 participants; they reported that circulating 25(OH)D levels were significantly lower in patients with chronic periodontal disease than in healthy controls (pooled mean difference: −2.05, 95% confidence interval: −3.40 to −0.71). Importantly, meta-analyses could not be conducted in most published systematic reviews because relatively few studies were available for inclusion.

In 2020, Millen and Pavlesen reviewed studies that evaluated an association between vitamin D and periodontal disease; some prospective studies implied that vitamin D prevents tooth loss [55]. In a scoping review by Diachkova et al., some analyzed studies showed an association between low vitamin D levels and the severity of periodontal disease, defined as either lower soft tissue attachment/attachment loss, changes in probing depth, or reduced indications of systemic inflammation [56].

## 5. Sex Differences in Vitamin D and Calcium Supplementation

### 5.1. Investigations of Sex Differences in Supplementation

Sex differences in status and supplementation have been reported for both calcium and vitamin D [21,22,23,24]. The studies have included a nationwide survey of Japanese adults aged ≥65 years [21], a survey of patients (most aged ≥50 years) in the St. Louis (MO, USA) region [22], a nationwide review of clinical records for patients aged ≥40 years in the United States [23], and a limited survey of patients (most aged ≥49 years) in the cities of Jeddah and Makkah (Saudi Arabia) [24]. Dixon et al. [22] studied patients with periodontal disease who participated in a disease maintenance program at two separate institutions, where no advice was provided concerning calcium or vitamin D intake; the authors found that women used significantly more calcium supplementation, compared with men (*p* < 0.001). However, the differences between women and men regarding vitamin D supplementation were not statistically significant.

Lee et al. [23] used national-level health data from the USA to evaluate 10-year trends in supplementation from 2000 to 2009; they found that female sex was a strong predictor of vitamin D and calcium supplementation (*p* < 0.0001). In a study performed in Saudi Arabia, Aljefree et al. [24] performed in-person interviews using semi-structured questionnaires and thematic analysis; compared with men, women reported greater interest in improving their vitamin D levels.

### 5.2. Interactions of Pregnancy with Periodontal and Systemic Health

Pregnancy is associated with periodontal disease [57]; women experience worsening of existing periodontal disease or develop periodontal disease during pregnancy [25,58]. In a review, Ortiz-Sánchez et al. [25] suggested that hormonal treatment, the use of hormonal contraceptives, and pregnancy can induce clinical, cytological, or microbiological changes in women that may promote the development of periodontal disease. In the same review, the authors stated that the immune response during pregnancy is modified through the effects of pregnancy-related hormones, which could influence the development of periodontal disease. Furthermore, the authors stated that the hormonal profile during pregnancy favors the accumulation of *Prevotella* in the subgingival microbiome, such that its abundance is up to 55-fold greater in pregnant women than in non-pregnant women.

Other studies have revealed associations of maternal periodontal disease with preterm birth [26] and/or low birth weight [27,28], which increases the neonatal mortality rate [29]. Chambrone et al. [27] found a consistent association between maternal periodontal disease and preterm birth and/or low birth weight. Similarly, Teshome and Yitayeh [28] reported a potential association between preterm birth and/or low birth weight in nine studies that the authors reviewed.

### 5.3. Vitamin D and Calcium Supplementation in Pregnancy

Vitamin supplementation in pregnancy is reportedly necessary to achieve a sufficient vitamin D status [59]. Multiple studies have revealed lower serum levels of vitamin D in pregnant women [60,61,62]. In the United States, Boggess et al. [60] found that the adjusted odds ratio (95% confidence interval) for moderate to severe periodontal disease among pregnant women with vitamin D insufficiency was 2.1 (0.99–4.5). To address this need for supplementation, two recent studies in Brazil evaluated milk fortification with vitamin D and calcium in low-income pregnant women with periodontal disease. The first study was a feasibility study [63] in which the authors evaluated the acceptability, adherence, and retention of milk fortification with vitamin D and calcium. The authors reported no adverse effects; the intervention was acceptable, well tolerated, and feasible in the study population of low-risk pregnant women. The second study was a related trial that evaluated several combinations of calcium and vitamin D supplementation in milk, in addition to early (during pregnancy) vs late (after delivery) periodontal therapy [64]. The authors found no differences among groups regarding fortification; however, early periodontal therapy was associated with significantly less bleeding on probing. The authors suggested that milk fortification may not have appeared to affect bleeding on probing because all women in the study drank milk; milk consumption is associated with fetal growth regardless of micronutrient fortification.

In addition to milk fortification, other interventions are available to improve vitamin D and calcium status during pregnancy. Potential public health interventions include advocating for vitamin D and calcium supplementation among women to avoid corresponding deficiencies and an increased risk of periodontal disease. Vitamin D impacts oral health; therefore, oral health can be improved by measuring vitamin D and supplementing as needed. Vitamin D levels should also be measured before oral therapy to ensure that the best outcomes are achieved.

Public health interventions to educate women on the sources of vitamin D and appropriate supplementation may also be beneficial. Such interventions could provide information about balancing sufficient sun exposure with skin cancer risk, ensuring sufficient dietary intake vitamin D (i.e., which foods are good sources of vitamin D and how much of each is needed to achieve adequate supplementation), and balancing sufficient sun exposure with sufficient oral supplementation.

## 6. Future Directions

Vitamin D and calcium supplementation can have a positive effect in the management of periodontal disease, and supplementation can be used as an adjunct to non-surgical periodontal treatment; however, the appropriate doses of vitamin D and calcium are currently unknown. The supplementation doses depend on the season (more vitamin D supplementation is required in winter [65]), and whether the study group comprises healthy, younger adult populations vs populations of people with illness. Thus, there is a need for investigations of vitamin D levels, as well as the required amounts of vitamin D and calcium supplementation, in specific populations and across seasons.

It remains unclear whether higher doses of vitamin D are more beneficial or less beneficial. In appropriate doses, vitamin D is generally considered safe, although excessive doses can be harmful [66]. As such, the maximum supplemented dose of vitamin D, whether for periodontal disease specifically, or as supplementation for other diseases, will depend on the initial measured level of 25(OH)D.

There is a need for consensus guidelines that recommend the measurement of 25(OH)D levels before periodontal surgery. Furthermore, vitamin D deficiency impacts oral health. Public health strategies are needed to increase vitamin D and calcium supplementation in pregnant women; vitamin D levels should also be assessed to ensure balanced oral health in this population. Specifically, vitamin D levels should be verified before the treatment of any oral conditions to achieve better patient outcomes. Furthermore, vitamin D helps to manage calcium–phosphate metabolism and bone regeneration. Periodontal surgery is usually associated with regenerative periodontal therapy; bone growth may be impeded by low levels of vitamin D.

## 7. Conclusions

Periodontal disease is common; affected individuals can benefit from supplementation with both vitamin D and calcium. However, there are barriers to the achievement of adequate vitamin D supplementation. These barriers may include lifestyle changes that result in less sun exposure and less ingestion of vitamin D-containing food than in previous generations. Barriers may also include confusion regarding vitamin D (i.e., benefits, possible need for supplementation, and methods for achieving adequate levels). Furthermore, there is insufficient knowledge about the appropriate dose of vitamin D for oral supplementation. Clear public health messaging is needed regarding the sources and effects of vitamin D, including specific information about vitamin D production in the skin.

There are sex differences in vitamin D supplementation; for example, more women show an interest in and are more inclined to improve their vitamin D levels, compared with these attitudes in men [24]. There are also sex differences in calcium status. Ishikawa et al. [21] showed that adults aged ≥75 years have greater deficiencies in protein, potassium, and calcium, and greater excesses of sodium, compared with adults aged 65–74 years. The authors reported that within-individual variance in calcium intake in men (*p* = 0.008) considerably decreased with age, while the proportion of women with calcium deficiencies increased with age. However, more studies are needed concerning the differences between women and men with respect to knowledge of vitamin D and calcium. Studies are also needed concerning the sex differences in the willingness to engage in vitamin D and calcium supplementation.

Periodontal disease may worsen during pregnancy [25]; vitamin D and calcium supplementation may help to reduce the adverse neonatal outcomes associated with periodontal disease. Without increasing the burden on public health resources, methods should be developed to help improve knowledge among pregnant women regarding the possible need for vitamin D and calcium supplementation.

Future research must address the gaps in knowledge regarding how sex affects the knowledge and supplementation of vitamin D and calcium. This may be addressed by designing specific public health policies to improve women’s knowledge regarding vitamin D and calcium supplementation; it may also be improved by increasing intakes of vitamin D and calcium. The results of implementing such policies should be analyzed via population-level knowledge assessment. They should also be analyzed by investigations of relevant physiological parameters and clinical outcomes. Additional research regarding the dose-related anti-inflammatory effect of vitamin D is needed to determine the ideal dose and to further define the anti-inflammatory effects, especially in relation to periodontal disease.

In summary, vitamin D and calcium have key roles in bone regeneration, which has a direct effect on periodontal disease. It is critical to recognize the importance of achieving adequate levels of both factors in women, especially pregnant women.

## Data Availability

Not applicable.
